# Exercise blood pressure, cardiorespiratory fitness, fatness and cardiovascular risk in children and adolescents

**DOI:** 10.3389/fpubh.2024.1298612

**Published:** 2024-06-13

**Authors:** Zhengzheng Huang, Xiuping Li, Xia Liu, Yayun Xu, Haixing Feng, Lijie Ren

**Affiliations:** ^1^Guangdong Key Laboratory for Biomedical Measurements and Ultrasound Imaging, National-Regional Key Technology Engineering Laboratory for Medical Ultrasound, School of Biomedical Engineering, Shenzhen University Medical School, Shenzhen, China; ^2^Department of Neurology, The First Affiliated Hospital of Shenzhen University, Shenzhen Second People's Hospital, Shenzhen, China; ^3^Department of Neurology, Shanghai General Hospital, Shanghai Jiao Tong University School of Medicine, Shanghai, China

**Keywords:** exercise blood pressure, masked hypertension, adolescence, hypertension, cardiorespirarory fitness

## Abstract

Cardiovascular disease remains the leading cause of mortality on a global scale. Individuals who possess risk factors for cardiovascular disease, such as high blood pressure (BP) and obesity, face an elevated risk of experiencing organ-specific pathophysiological changes. This damage includes pathophysiological changes in the heart and peripheral vascular systems, such as ventricular hypertrophy, arterial stiffening, and vascular narrowing and stenosis. Consequently, these damages are associated with an increased risk of developing severe cardiovascular outcomes including stroke, myocardial infarction, heart failure, and coronary heart disease. Among all the risk factors associated with cardiovascular disease, high blood pressure emerges as the most prominent. However, conventional resting BP measurement methods such as auscultatory or oscillometric methods may fail to identify many individuals with asymptomatic high BP. Recently, exercise BP has emerged as a valuable diagnostic tool for identifying real (high) blood pressure levels and assessing underlying cardiovascular risk, in addition to resting BP measurements in adults. Furthermore, numerous established factors, such as low cardiorespiratory fitness and high body fatness, have been confirmed to contribute to exercise BP and the associated cardiovascular risk. Modifying these factors may help reduce high exercise BP and, consequently, alleviate the burden of cardiovascular disease. A significant body of evidence has demonstrated cardiovascular disease in later life have their origins in early life. Children and adolescents with these cardiovascular risk factors also possess a greater propensity to develop cardiovascular diseases later in life. Nevertheless, the majority of previous studies on the clinical utility of exercise BP have been conducted in middle-to-older aged populations, often with pre-existing clinical conditions. Therefore, there is a need to investigate further of the factors influencing exercise BP in adolescence and its association with cardiovascular risk in early life. Our previously published work showed that exercise BP is a potential useful method to detect adolescents with increased cardiovascular risk. Children and adolescents with cardiovascular risk factors are more likely to develop cardiovascular diseases later in life. However, previous studies on the clinical utility of exercise BP have largely focused on middle-to-older aged populations with pre-existing clinical conditions. Therefore, there is a need to investigate further the factors influencing exercise BP in adolescence and its association with future cardiovascular risk. Our previous studies, which focused on exercise BP measured at submaximal intensity, have shown that exercise BP is a potentially useful method for identifying adolescents at increased cardiovascular risk. Our previous findings suggest that improving cardio-respiratory fitness and reducing body fatness may help to reduce the risk of developing cardiovascular disease and improve overall cardiovascular health. These findings have important implications for the development of effective prevention and early detection strategies, which can contribute to improved public health outcomes.

## Introduction

Cardiovascular disease is the leading cause of death worldwide, with individuals who have risk factors like high blood pressure (BP) and obesity being more likely to develop organ-specific pathophysiological changes that can lead to severe outcomes such as stroke, heart failure, and ischemic heart disease. High BP is identified as the most significant risk factor among all (1–3). Whilst this remains true, conventional resting BP measurement methods may fail to detect asymptomatic hypertension (4). Exercise BP has recently been proven useful in revealing high BP and underlying cardiovascular risk in complementary to resting BP in adults (5–7). Moreover, multiple factors such as cardiorespiratory fitness and fatness have also been revealed to contribute to exercise BP and related cardiovascular risk (8). Modifying these factors might lower high BP response to exercise and, as a result, alleviate the burden of cardiovascular disease. Of note, substantial evidence has shown that cardiovascular diseases in later life have an early life origin and children. Children and adolescents with these cardiovascular risk factors have a greater propensity to develop cardiovascular diseases in later life (9). Nonetheless, most of the previous studies on the clinical utility of exercise BP were conducted in the middle-to-older aged population often with pre-existing clinical conditions (8–10). Standardized exercise protocols are employed in specific clinical studies, as well as for evaluating the cardiorespiratory fitness. However, the limited availability of reference values for exercise BP in children and adolescents under various protocols complicates the interpretation of BP readings in these populations. Therefore, there is a need to gain deeper insights into the contribution of the factors on exercise BP in children and adolescence, and explore the association between exercise BP thresholds and long-term cardiovascular risk for developing effective prevention and early detection strategies.

The overarching aim was to summarize how these factors (such as cardio-respiratory fitness and fatness) contribute to exercise BP and its related potential cardiovascular risk in children and adolescence. We also want to provide an overview of the concept of exercise BP and related cardiovascular risk and to discuss essential literature on the clinical relevance of exercise BP, evidence of the early life origin of cardiovascular disease and rationale for utilising exercise BP in childhood and adolescence in revealing cardiovascular risk.

### Cardiovascular disease risk factors

Many risk factors play an important role in contributing to the development of cardiovascular disease. These risk factors include modifiable factors such as high BP, diabetes, abnormal blood lipids, obesity, unhealthy diet, physical inactivity and socioeconomic status, and the non-modifiable risk factors of age and family history. Of all the identified risk factors, high BP stands out as the predominant risk factor for cardiovascular disease (11). A meta-analysis showed that for each 10 mm Hg reduction of SBP, the risk of major cardiovascular disease events including coronary heart disease (0.83, 0.78–0.88), stroke (0.73, 0.68–0.77), and heart failure (0.72, 0.67–0.78) are substantially reduced (12). The reduction of 10 mmHg of SBP has been shown to lead to a significant 13% reduction in all-cause mortality (0.87, 0.84–0.91) (12). Therefore, by managing high BP, the burden of cardiovascular disease can be significantly reduced.

## Blood pressure – the greatest risk factor for cardiovascular disease

### What is (high) blood pressure?

The heart provides tissues and organs of the human body with oxygenated blood for sustaining life (13). With each heartbeat, blood is pumped into the blood vessels of the circulatory system, the flow of which generates a perpendicular force against the endoluminal surface of the arterial walls (13). This force is termed arterial BP (13). BP is typically viewed as two numbers: the highest number being systolic pressure, representing the peak of pressure against the arterial wall during the contraction phase (14). Diastolic pressure is the BP in the arteries during the relaxation phase (14). In clinical practice, both systolic and diastolic BP are generally recorded with a unit of millimetres of mercury (mmHg) (14).

High BP, also known as hypertension, is a condition in which the pressure on the arterial walls (systolic and/or diastolic BP) is higher than normal (15). There is a continuum of risks related to BP, although high BP is classified according to a threshold level in clinical practice (16–19). Thresholds used to define high BP differ among regional clinical guidelines. For instance, the European guidelines define high BP as the average of more than two BP readings above 140 mmHg for SBP or above 90 mmHg for diastolic BP on more than two occasions (18). The same definitions are used for the ‘in-office’ hypertension in the 2020 International Society of Hypertension global hypertension practice guidelines (17). However, the US guidelines propose a lower threshold of 130 for systolic and 80 mmHg for diastolic since 2017 (16). Despite the differences in thresholds for defining high BP, guidelines are in agreement on the necessity of taking multiple measurements and including the provision of out-of-office BP measurements such as ambulatory BP monitoring (ABPM) or home BP monitoring (HBPM) to confirm true BP level for clinical decisions (16–19). Diagnosing high BP/hypertension in pediatric and adolescent populations poses a significant challenge due to the complexity of standards and definitions during periods of rapid growth which makes it difficult to design outcome cardiovascular studies. Despite the heterogenity of definiton in different countries, high BP/hypertension in children and adolescents is usually defined as values exceeding the 95th percentile of age-specific and sex-specific distributions (20, 21).

### How is BP measured and managed in clinical practice?

Despite the high prevalence of high BP and advancements in BP measurement and monitoring, there is still a lack of identification and control of high BP. According to WHO, approximately 46% of the adult population with high BP are unaware of their diagnosis (1). Given the significant role of high BP in contributing to cardiovascular risk, accurate measurement of BP in clinical practice is paramount (11, 22, 23). It is important to follow clinical guidelines for BP measurement to avoid misclassification of cardiovascular risk and potential adverse health consequences (23).

In real practice, health professionals typically measure BP in a clinic setting using a manual auscultatory or automatic oscillometric device that has been validated according to standardized protocols, known as “office BP” or “clinic BP” (24). Accurate measurement of BP in clinical practice is essential for assessing cardiovascular risk. To address the “white-coat effect, “where BP rises in the presence of health professionals, unobserved office BP measurements should be taken using an automatic device while patients sit alone in the doctor’s office. These unobserved office BP readings are typically lower than conventional office BP readings, (25) but similar to or lower than average daytime BP readings measured by ambulatory blood pressure monitoring (ABPM), which is considered as the gold-standard BP measurement (26).

In addition to office BP measurements, out-of-office BP measurement is commonly performed in clinical practice. This refers to BP measurements taken outside of the doctor’s office, such as at home or in the workplace, and is considered more reflective of daily life BP. Home BP monitoring (HBPM) is usually done by the patient using an automatic oscillometric device in a quiet environment at home in the morning. HBPM measurements are typically lower than office BP and showed higher reproducibility (27). HBPM has also been found to be more closely associated with targeted organ damage, such as left-ventricular hypertrophy, compared to office BP (27).

Another form of out-of-office BP measurement is ABPM, which is considered the gold-standard method to confirm high BP (27). ABPM records multiple BP readings over a 24-h period using a portable automatic oscillometric BP monitor. ABPM is more closely associated with hypertension-mediated organ damage and cardiovascular mortality than office BP (27, 28). Since the ABPM device is expensive and the side effects includs sleep interference, plan, skin irritation, noisy device, inconvenience with work, haematoma etc. for some patients (29). ABPM can not be performed without specialist equipment and clinical supervision. Thus, ABPM might not be appropriate for screening high BP in public health settings or in large population-based studies where there are time and budget constraints. Thus, there is a practical need to find other alternatives for population detection and screening for high BP.

In clinical practice, treatment decisions for high BP should consider factors beyond BP. Both US and European guidelines recommend considering coexisting risk factors to reduce underlying cardiovascular risk (16–18). High BP rarely exists alone, and risk factors that coexist with high BP have a multiplier effect on the risk of cardiovascular disease (30). Previous evidence suggests that cardiovascular risk calculator identifies patients at high risk who may not be identified through traditional BP measurement. Identification of individuals at high risk of developing cardiovascular risk based on algorithms which integrate several cardiovascular risk factors has been shown to be more effective than targeting BP alone (30, 31). Quantifying future cardiovascular risk in patients with hypertension is particularly important for healthcare service and disease prevention.

### What are the current shortfalls in the current measurement and management of high BP?

Although the prevalence of high BP remains high worldwide, many asymptomatic individuals are unaware of their high BP diagnosis. Although office BP measurements are frequently utilized in clinical settings, they may not consistently provide an accurate representation of an individual’s true BP status. White-coat hypertension is characterized by elevated BP readings obtained in a clinical setting that exceed the recommended thresholds, whereas measurements obtained through HBPM or ABPM demonstrate normotensive values. This observation indicates that the increased office BP may be a phenomenon triggered by the presence of a health professional. On the other hand, office BP measurements can also underestimate a person’s true BP level, leading to a condition known as masked hypertension (18–20). This occurs when BP readings are normal in the clinic but elevated outside of the office on HBPM or ABPM. Individuals with masked hypertension have an increased cardiovascular risk compared to those with normal BP (30). Nevertheless, this risk is frequently overlooked due to the absence of a recommendation for ABPM when an individual’s office BP falls within the normal range. For patients who are suspicious of masked hypertension, current guidelines recommend confirming BP levels with HBPM or ABPM rather than relying solely on a single clinical BP (16–19). Nevertheless, these individuals are frequently overlooked as a result of their “normal” clinic BP, and ABPM may not always be feasible for patients who struggle with wearing the monitoring device due to discomfort in the arm and disturbances in sleep (29). Therefore, other alternative methods are needed to identify masked hypertension and its associated risks.

## Exercise BP

### The acute BP response to exercise

Dynamic exercise increases the metabolic demand for skeletal muscles (32). Coordinated physiological responses to exercise fulfil this physiological need of the active musculature whilst maintaining sufficient blood supply to vital organs such as the heart and brain (33). Peripheral resistance reduces as a result of redistribution of blood flow from non-activated to activated circulatory beds and muscular arteriole dilates in order to supply skeletal muscle (34). Moreover, cardiac output increases due to upregulated sympathetic activity and accelerated heart rate as the workload of exercise increases (35). The increase in cardiac output predominates over the decrease in systemic vascular resistance, which can lead to as much as a 40% rise in mean arterial pressure (33). Regardless of the mode of the exercise test, systolic BP normally increases with exercise intensity and reaches its peak at maximal exercise intensity/capacity, (36) whilst diastolic BP remains largely unchanged (37). Although there is no unanimity on the definition of normal BP response to exercise, some studies used absolute value. For instance, according to Framingham criteria, a peak systolic BP reaching 210 mmHg for males and systolic BP reaching 190 mmHg for females was considered as the theoretical maximum values for treadmill testing according to standard protocols (often the Bruce protocol) (38, 39). In contrast, in other studies, maximal systolic BP below the 90th or 95th percentile of the studied population was considered normal (33, 34, 40). It is worth noticing that the peak exercise systolic BP, diastolic BP and the increase in systolic BP from rest to peak intensity were higher in men than in women in the same age group and the peak exercise systolic BP was shown to be positively associated with age (33, 41). Therefore, both gender and age should be taken into consideration when interpret BP responses to exercise.

### Abnormal BP response to exercise

Despite the majority of people who undertake an exercise test presenting a normal BP response, some individuals may demonstrate an abnormal BP response to exercise, either a hypotensive response or a hypertensive response. Hypotensive response to exercise is a transient drop below the pre-test resting BP or failure of an increase of 10 mmHg in pre-test resting systolic BP as the exercise intensity progressively increases (42, 43). Currently there is no consensus on the magnitude of BP decrement or duration of the response (33, 42). Individuals with borderline hypertension have been reported as more likely to experience a greater drop in post-exercise BP than those without high BP (44, 45). A hypotensive response to exercise such as a decrease in systolic BP of 10 mmHg or more with the increased workload is an indication for termination of exercise testing (44, 46). The incidence of hypotensive response to exercise was previously reported 3 to 6% among those being referred to treadmill exercise testing (42, 47–49). Hypotensive response has been reported not only associated with a higher prevalence of coronary artery disease or left ventricular dysfunction but also a worse prognosis of cardiovascular events in those individuals with or without established cardiovascular diseases (42, 47–51). Hypotensive response to exercise was thought to be attributed to a decreased cardiac output and systemic vascular resistance, altered venous return and/or myocardial contractility, however, the underlying cause of abnormal haemodynamics has not yet been revealed and therefore warrants further investigation (44, 48, 52). Whilst hypotensive response is associated with cardiac dysfunction and subsequent cardiac events, it is not the focus of this review.

**Figure 1:**
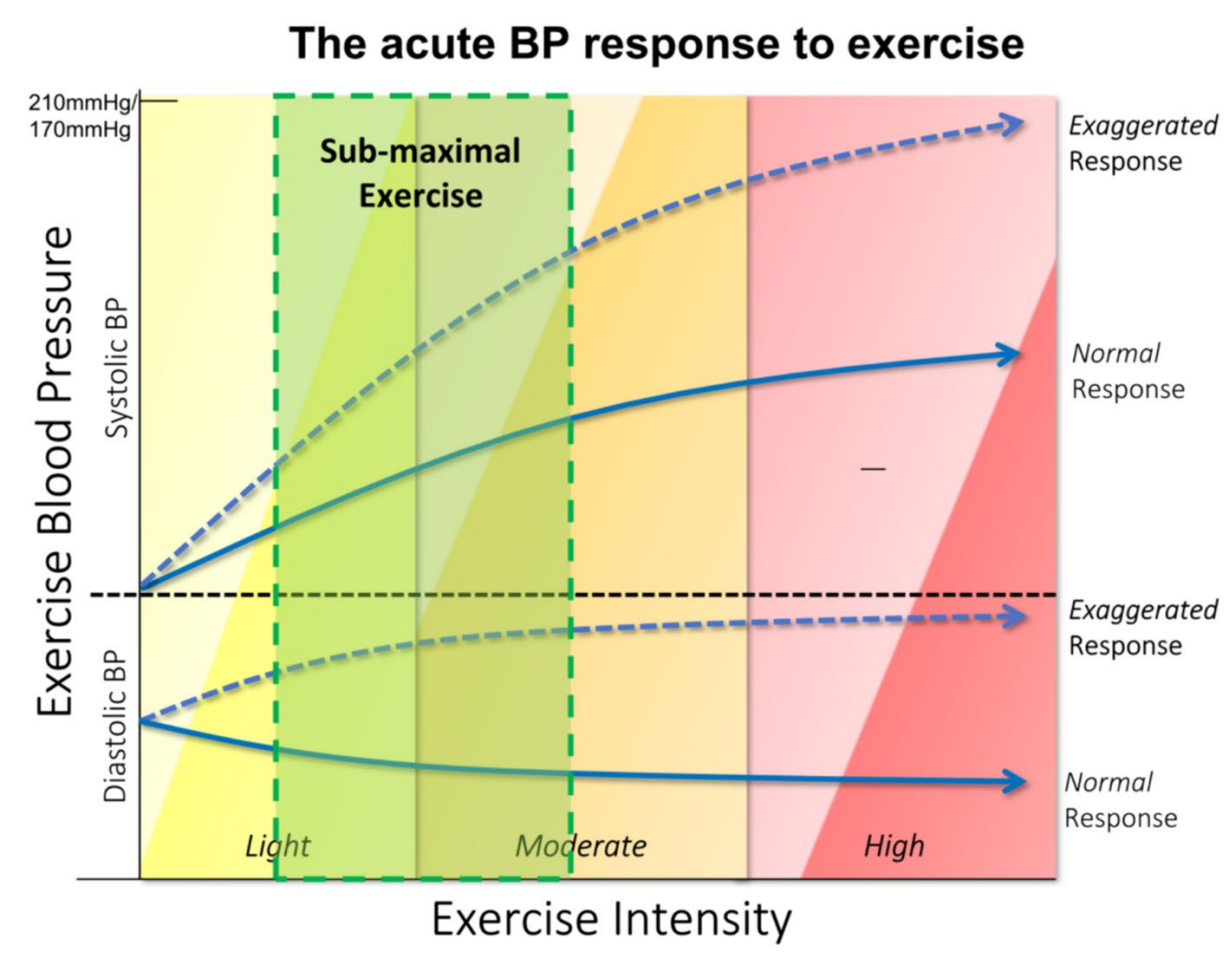
Exaggerated exercise BP or hypertensive BP response to exercise.

In contrast with hypotensive BP response, an excessive rise in exercise systolic BP is termed as a hypertensive response to exercise or exaggerated exercise BP (Figure 1), although the pathophysiological mechanism behind this rise remains unclear (48). Exaggerated BP response to exercise can occur at any stage or different intensity of exercise and in both a healthy population and patients on antihypertensive medications (38–41, 48). Exaggerated exercise BP measured at a fixed light or moderate intensity has been revealed to have higher prognostic value in predicting future cardiovascular risk than resting BP or exercise BP measured at maximal intensity, (37, 48) although currently there is no definitive threshold to define exaggerated exercise BP at either submaximal or maximal intensity of exercise. Some studies utilized specific BP thresholds to define an exaggerated exercise BP while others used the 90th to 95th percentile of the BP response in the studied population. For instance, a systolic BP of 210 mmHg in males or a systolic BP of 190 mmHg in females was adopted as the cut-off values for exaggerated exercise BP at a maximal intensity of exercise in several studies including the Framingham Heart Study (49–52). According to the ACSM guideline, a peak systolic BP greater than 250 mmHg or diastolic BP greater than 115 mm Hg with maximal exercise testing is defined as an exaggerated exercise BP (43). In addition to an exaggerated exercise BP at the peak or maximal exercise intensity, various thresholds ranging from 150 to 180 mmHg have been adopted at submaximal intensity in relation to different cardiovascular outcomes with different intensity and protocols of exercise tests (8). However, in the case of adults, the determination of elevated exercise BP is hindered by variations in testing methods and protocols, such as whether the exercise is conducted on a treadmill or bicycle, and whether BP is measured during moderate or maximum intensity. As mentioned in the hypertension guidelines, the definition of elevated BP in children is based on sex-and age-specific reference values rather than fixed cut-off points. The scarcity of studies investigating exercise BP in the children/adolescent population complicates the interpretation of exercise BP in these groups. A recent published review advised that (1) the external exercise workload remain consistent when measuring exercise BP, and that (2) exercise BP should be assessed at a fixed submaximal exercise intensity (8). These aforementioned suggestions warrant a proper interpretation of exercise-induced BP changes in relation to cardiorespiratory fitness, and can be easily incorporated into practice. Further investigation is needed to establish standard reference values and assess the clinical relevance of indexing exercise systolic BP responses in diverse and extensive populations.

Whilst the mechanism behind exaggerated exercise BP remains to be elucidated, abnormal haemodynamics may be a key factor that contributes to the excessive rise in exercise BP. Previous studies suggested that impaired vascular function which includes increased arterial stiffness and impaired endothelial function is associated with exaggerated exercise BP (51, 53). This association between impaired vascular function and exaggerated exercise BP may explain the mechanism link between exercise BP and increased cardiovascular risk. A previous study also indicated that those with exaggerated exercise BP may have an underlying impaired vascular function (51). The vascular system with impaired vascular function fails to compensate for the increased cardiac output caused by daily normal physical activities and therefore leading to frequent rises in BP (35).

The dashed line in this figure depicts the excessive rise of the systolic BP in response to the increasing intensity with the progression of the exercise test. This abnormal rise which is called an exaggerated exercise BP or a hypertensive BP response to exercise occurs in some individuals, irrespective of their normal resting BP. The solid line represents the normal increase of exercise BP which is termed as a normal BP response.

## Clinical relevance of abnormal exercise BP

### Incident hypertension

Over the past four decades, increasing evidence has emerged suggesting that exaggerated BP response to exercise is complementary to conventional office BP in predicting incidents of hypertension among apparently healthy populations (54–57). For instance, the prognostic value of an exaggerated exercise BP measured at a graded treadmill test was reported higher in predicting the future incidence of hypertension than resting BP in general populations with normal resting BP (54). In a longitudinal study of 3,741 apparently healthy middle-aged men with normal office BP, those whose peak exercise systolic BP greater than 181 mmHg had a 1.54-fold higher risk of developing hypertension compared to those whose peak exercise BP was below the cut-off (50). This peak exercise systolic BP cut-off was also shown to be the strongest predictor of future hypertension (50). Similarly, another study of 1,534 middle-aged male participants with normal resting BP also showed that participants with peak exercise systolic BP greater than 200 mmHg would develop a higher resting BP after 10 years (58). Moreover, the baseline exercise systolic BP was confirmed a stronger predictor of future systolic BP than baseline resting BP after an average of a 10-year follow-up (58).

Due to the differences in mode of exercise and choice of protocol, different threshold values are used to define exaggerated exercise BP in adults, showing a notable variation. When exercising at maximum intensity, the 90th-95th percentiles of exercise BP specific to each gender (usually corresponding to 210/110 mmHg or higher for males and 190/110 mmHg or higher for females) are commonly employed to indicate exaggerated exercise BP (57, 59). Consistent with the aforementioned data, a peak exercise systolic BP of 210 mmHg for males or 190 mmHg for females was established as the cut-off to predict the incidence of hypertension 5 years later in individuals with normal resting BP (57). In addition to exercise BP measured at peak intensity, a submaximal exercise BP cut-off of 160 mmHg at 100 W workload of a symptom-limited cycle ergometry test for males was reported as the optimal cut-off value in predicting the incidence of hypertension independently of other established cardiovascular risk factors in normotensive males (59).

The utility of exaggerated exercise BP in predicting the development of hypertension has been confirmed in specific populations such as young athletes (60). For instance, a young athlete with an exaggerated BP response to exercise defined as peak exercise BP greater than 220/85 mmHg for males or 200/80 mmHg for females had a 3.6 times higher chance of developing into hypertension than an athlete with a normal exercise BP response (60). Several meta-analyses also demonstrated that an exaggerated exercise BP is associated with future cardiovascular risk irrespective of exercise mode/intensity (10, 54).

### Underlying high BP/masked hypertension

Instead of staying in a resting state for an entire day, individuals spend substantial time each day in an ambulatory state. The major concern with only applying clinic (office) BP measurement is that it fails to capture high BP which occurs out of the office (masked hypertension) (7). For those with normal in-clinic BP but raised out-of-office BP, there is no indication for them to be referred to do an out-of-office BP measurement (ambulatory BP monitoring). Therefore, the hidden cardiovascular risk of masked hypertension can be easily missed if individuals are only provided with a conventional office BP measurement. Thus, there is a practical need to find other methods to detect those with underlying high BP such as masked hypertension. BP is measured during a submaximal (light-to-moderate) intensity exercise which is representative of the physiological response to daily life physical activity and it correlates well with ambulatory BP monitoring (51, 61). Thus, BP measured during submaximal exercise may be a more might be more reflective of the BP in daily life than BP measured in the resting state in the clinic.

Given the high prevalence of masked hypertension (58%) among those with a hypertensive response to exercise BP, many studies suggested that exercise BP might be a useful tool in revealing masked hypertension and related cardiovascular risk (5, 7, 61, 62). For instance, peak exercise systolic BP has been confirmed related to underlying high BP missed by office BP but also related to underlying high BP when measured by ABPM (63). Although most of the previous studies of exercise BP only focus on peak or maximal intensity exercise BP, submaximal intensity exercise is similar to daily physical activity. Therefore, various thresholds of BP response to submaximal intensity exercise have been revealed associated with masked hypertension in adults (5, 7, 61–63). For instance, a systolic BP above 150 mmHg at an early stage of exercise stress testing was reported to reveal masked hypertension in individuals with apparent normal office BP (5). Similarly, an exercise systolic BP greater than 175 mmHg at low intensity was reported to reveal masked hypertension with high sensitivity (74%) and moderate specificity (67%) (7). These findings could be partially explained by the fact that the BP response to moderate-intensity exercise is more akin to the chronic BP load that people encounter in their daily physical activity and therefore more reflective of ‘true BP’ (8, 9). Taken together, all these results suggested that exaggerated exercise might be a warning signal of underlying high BP (masked hypertension) and related cardiovascular risk which is undetectable through conventional resting BP measurement.

### Factors associated with exercise BP

Exercise BP has been shown associated with many factors such as increasing age, (30, 64) gender, (65) cardiorespiratory fitness (8, 48), and insulin sensitivity (55). Among all these factors, cardiorespiratory fitness is not only an important modifier of exercise BP but also a powerful predictor of future cardiovascular events and mortality (8, 57, 63, 66).

### Cardiorespiratory fitness

Cardiorespiratory fitness is known as a clinical indicator to reveal the body’s capacity for transport and consumption of oxygen during aerobic exercise and is traditionally represented by indices such as maximum oxygen uptake, physical work capacity, and resting heart rate. Cardiorespiratory fitness is considered an important cardiovascular risk factor since low cardiorespiratory fitness has been shown associated with higher all-cause and cardiovascular mortality (67–69). Moreover, high cardiorespiratory fitness has also been confirmed associated with a higher level of physical activity, which in turn creates more health benefits such as lower occurrence of coronary heart disease (39). Whilst numerous methods can be used to assess cardiorespiratory fitness either directly or indirectly, the gold standard or most objective measure of cardiorespiratory fitness is maximal oxygen uptake (VO_2_ max). An individual’s maximum capacity of oxygen utility (VO_2_ max or peak VO_2_ more appropriate) is normally assessed in a graded exercise treadmill or cycling test by wearing a ventilation mask and VO_2_ max was generated by analysing the concentrations of oxygen and carbon dioxide of inspired and expired air (70–72). Other methods such as an indirect estimation of VO_2_ max generated from validated metabolic equations are also acceptable under circumstances where a direct measure is not applicable (73–75).

The health benefits of improvement in cardiorespiratory fitness have been reported by many clinical studies. Substantial evidence showed that low cardiorespiratory fitness is associated with increased all-cause mortality from many causes, especially from cardiovascular disease (57, 63). Accordingly, improvement of cardiorespiratory fitness provides a variety of health benefits such as decreased incidence of cardiovascular diseases and mortality and better prognosis of cardiovascular events (51, 57, 63, 76). Cardiorespiratory fitness has been reported to modify exercise BP in different populations irrespective of resting BP. Previous studies indicated that exercise BP is negatively associated with cardiorespiratory fitness such that individuals with high fitness levels would exhibit a lower exercise BP compared to peers with lower fitness levels (76). Moreover, this negative linear association between exercise BP and cardiorespiratory fitness has been found in both normal, prehypertension, and hypertensive populations (72, 75). Furthermore, longitudinal studies have also shown that higher cardiorespiratory fitness at baseline was associated with lower submaximal systolic BP after years of follow-up in an apparently healthy middle-aged population (57).

Although the mechanism behind how cardiorespiratory fitness modifies exercise BP remains unclear, it can be speculated that exercise training improves the efficiency of oxygen delivery and consumption and therefore leads to an increase in fitness (63). For individuals with improved fitness, the cardiac work during exercise is much reduced as a result of a lower heart rate at a fixed cardiac output (51). As a result, a decrease in exercise BP was observed in adults who underwent lifestyle interventions such as exercise training (75).

Indeed, those with low cardiorespiratory fitness usually exhibited a high exercise systolic BP, however, a high exercise BP response could also be seen in those with high fitness. For instance, a reverse J-shape relationship between fitness and exercise systolic BP was reported in a cohort of young males such that a high exercise BP was found in those with low and high fitness (77). The high exercise BP exhibited in those with high fitness could be explained by the fact that cardiac output is the major determinant of fitness (78). Therefore, exercise BP increases due to high cardiac output associated with high fitness. Higher fitness has been shown conversely associated with a lower all-cause mortality rate due to the reduced rate of cardiovascular disease and cancer (51, 79). Thus, an elevation in exercise BP for those with high fitness might not share the same level of potential cardiovascular risk as those with low fitness. Moreover, an absolute value of exercise BP may not be adequate in assessing cardiovascular risk in the absence of cardiorespiratory fitness. Some recent studies suggested that indexing the increase in peak systolic BP to the increase in workload is more predictive of cardiovascular mortality than peak exercise systolic BP alone (80). These findings suggest the importance of combining fitness or workload with exercise BP in assessing exercise BP-related cardiovascular risk in practice.

### Body composition

Body composition is often used to describe the different components of the human body such as lean body mass and body fat mass etc. Many aspects of body composition including quantity and distribution of body fat mass, and lean mass, are considered as critical outcomes in infant’s and children’s health research (81). Obesity and the proportion of body fat are well-established risk factors for chronic diseases, especially cardiovascular diseases. Some anthropometric indices of body composition such as body mass index (BMI), skinfold thickness, and the waist/hip circumference ratio (WHR) are conventionally used indicators of obesity or fatness level (82). These indices of body composition are widely used to quantify or monitor the change in obesity status and to assess related cardiovascular risk at either individual or population levels.

Whilst some factors such as gender, ethnicity and age were recognized to have a huge influence on these indices of body composition and sometimes it will lead to a misclassification of obesity. Other limitations still exist in using these indirect measures of body composition. For instance, BMI is a convenient measure of weight relative to height. BMI estimates body fatness level only on two basic anthropometric components: height and weight (BMI = weight in kg/height^2^ in m^2^). BMI is widely used in epidemiological or clinical studies with a large population to assess the obesity level. However, there are some limitations in using BMI. It is not uncommon for individuals with well-developed muscles to be categorized as overweight according to their body mass index (BMI). However, this classification can be deceptive as it fails to account for the possibility that these individuals may possess minimal or no surplus body fat. Conversely, BMI has the potential to underestimate the level of adiposity in individuals with limited muscle mass, such as the older adults or those who are physically weak (83). What’s more important than misdiagnosis is that the same level of BMI at different ages consists of different proportions of fat and fat-free mass. Therefore, differences in the body composition and components of fat and fat-free mass cannot be clearly distinguished using BMI. A more objective measure for different components of body composition is available with technological advances. Dual-energy x-ray absorptiometry (DXA) is a precise measurement method for body composition as it differentiates bone mass, fat mass and lean mass and thus, the proportion of each component can be easily calculated (84).

Data from prospective studies have shown that individuals with increased fatness characterised by an increase in waist circumference, waist/hip circumference ratio (WHR), or sum of skinfold were at heightened risk for developing cardiovascular diseases and had a higher incidence of cardiovascular mobility and mortality (73, 85). For instance, high BMI and fatness were associated with increased occurrence of cardiovascular diseases such as coronary heart disease, ischemic stroke, hypertension, and diabetes mellitus (79, 86–88). Due to lifestyle changes such as a poor diet and lack of exercise, the prevalence of fatness or obesity has increased in general. The prevalence of obesity has been reported to double and even quadrupled over the last three decades (85). An increase in body fatness or obesity as a result of the complexity of genetic, socioeconomic, environmental, and behavioral factors has become a major health and socioeconomic burden for individuals, health systems and society. Moreover, an unabated rise in the prevalence of obesity is expected to continue in the future with a higher incidence of cardiovascular diseases (89). Thus, monitoring and control of obesity remain a major public health problem worldwide that needs to be prioritized (1).

Body composition including body fatness and leanness are known risk factors for cardiovascular diseases in general. More specifically, body fatness is an important risk factor for both BP measured at rest and during exercise (86–88). High body fatness has been revealed positively associated with exercise BP in adults (70). A growing body of evidence also suggested that lifestyle interventions such as exercise training would lead to an attenuated exercise BP through a reduction in weight or body fat (70, 76). The Previous study also showed that reduced fatness through exercise training would lead to reduced exercise BP (75). Whilst the previous study was done in older adults with established cardiovascular diseases, the association between body composition (body fatness and leanness) and exercise BP has been seldom explored in a healthy adolescent population. By addressing these questions in the following sections, the influence of body composition on exercise BP and related cardiovascular risk can be better understood. Understanding these associations would enable the implementation of more appropriate treatment and management strategies which help to lower the incidence of cardiovascular morbidity and mortality caused by high fatness/obesity among these populations.

### Exercise BP in adolescence

Given that high exercise BP appears to be associated with incident hypertension and cardiovascular disease in adults, it is crucial to detect underlying high BP using exercise BP and to identify the factors that modify exercise BP and its related cardiovascular risk. This could be especially important in early life such as childhood or adolescence since there is increasing evidence showing that high BP is established at an early time point in life as indicated in a meta-analysis where BP tracking from childhood to adulthood was strong (68).

While the association between exercise BP and the well-established cardiovascular risk factors has been confirmed in adult populations, (51) studies on these associations in early life in adolescence are scarce. For instance, decreased adiposity or body fatness through exercise training has been reported as related to a reduction in exercise BP in adults (75). Obese hypertensive adolescents have higher BP than lean hypertensives during exercise (90). Whilst lower submaximal exercise BP has been shown associated with higher cardiorespiratory fitness in the older adult population, (75) a reverse J shape association between exercise BP and fitness has also been reported in a cohort of young men (77). However, the mechanism or reason behind the J shape relationship between fitness and exercise BP in youth remains to be elucidated. Thus, more studies are needed to explore the association between exercise BP and fitness in adolescents.

Although it has been revealed that exercise BP is associated with altered cardiovascular structure and function in adults, (48) exercise BP in adolescence has also been shown associated with cardiovascular structure in adolescence (91). Moreover, less is known about whether exercise BP in adolescence is associated with an adapted cardiovascular structure or future cardiovascular risk. Currently, few large population-based studies have examined the long-term association between exercise BP and cardiovascular structure and future cardiovascular risk in healthy adolescents.

Our previous studies focused on the clinical importance of exercise BP in detecting high BP and the factors that contribute to exercise BP (fitness and fatness) and the influence of exercise BP on cardiovascular risk (high BP and altered cardiovascular structure) in adolescents (92–94). One of our previous studies first demonstrated that exercise BP might be a useful tool to rule out underlying high BP or “masked hypertension” in adolescence (Specificity: 86.9%, NPV: 95.5%) (92). Adolescents with exercise BP above the thresholds are more likely to have a worse left ventricular mass index and aortic PWV (92). Moreover, the influence of fitness on the association between post-exercise BP and LV mass index was ‘U shaped’, suggesting that both those with low and high cardiorespiratory fitness shared a similar association which highlights the importance of considering fitness as a factor that contributes to both exercise BP and its relationship with the cardiac structure in adolescence (93). In addition to cardiorespiratory fitness, fatness was also associated with post-exercise systolic BP and might have a relatively more important role in exercise BP in adolescence (94). Furthermore, three distinctive trajectories of fatness and leanness from childhood to early adulthood were identified (unpublished work). Those in trajectory with the consistently highest level of fatness or lowest level of leanness had higher post-exercise systolic SBP. However, these associations were largely attenuated after adjustment for adult measures of fatness or leanness, which suggested that modelling trajectories of fatness and leanness from childhood to adulthood does not provide additional information about exercise BP than a single concurrent measurement in adulthood. Taken together, this review has discussed the potential importance of fitness and to a greater extent, fatness in contributing to exercise BP in adolescence. Higher exercise BP appears to be associated with high BP and altered cardiovascular structure, (49) highlighting the potential clinical implications of assessing exercise BP in adolescents for the identification of current and future cardiovascular disease risks.

Although this review provided some new insights into the understanding of the role of exercise BP and its association with fitness and fatness in adolescents, there are important issues to be addressed in future research. Our previous study showed that specific thresholds derived from exercise BP from a submaximal exercise step-test has a high specificity and negative predictive value in predicting masked hypertension in adolescence (92). This result suggested that exercise BP may serve as a useful screening tool in complementary to clinic BP for ruling out masked hypertension and potential cardiovascular risk. For example, BP measurements can be incorporated as a customary examination in educational institutions during children’s physical education classes. This may involve conducting pre-and post-exercise tests at sub-maximal intensity levels. The measurement of BP prior to and following exercise can provide valuable insights into the presence of underlying hypertension, warranting further investigation through specialized examinations to confirm the diagnosis. Furthermore, it is worth noting that the sensitivity and positive predictive value exhibit relatively low values. Consequently, a persisting challenge lies in the identification of readily available BP measures that can effectively detect masked hypertension.

Apart from this, exercise systolic BP above these thresholds was associated with greater cardiovascular risk in adolescence. To extend the clinical value of these findings, these thresholds should be tested in further large-scale population-based studies to validate the prognostic efficiency for predicting future cardiovascular risk/events. Also, future studies should focus on specifying prognostic/diagnostic thresholds of exercise BP in children and adolescents (different gender/age at a (fix) external workload) to predict future clinical outcomes or cardiovascular events. Notwithstanding the constraints of the preceding investigations, the aforementioned findings emphasize the pragmatic efficacy of exercise BP as a complementary tool for identifying and promptly detecting latent hypertension and its correlated cardiovascular hazards among children/adolescents.

## Summary/conclusion

In summary, high exercise BP seems to be associated with underlying high BP and altered cardiovascular structure and increased cardiovascular risk, emphasizing the potential clinical utility of exercise BP in the assessment or identification of existing and future cardiovascular disease risk in adolescence.

High BP and related cardiovascular diseases add a huge social and economic burden to society as many individuals with asymptomatic high BP remained undiagnosed and uncontrolled. Thus, there is an urgent and practical need to find other alternatives to detect underlying high BP and cardiovascular risk. Despite previous evidence showing that exercise BP is useful in revealing underlying cardiovascular risk, underlying high BP or masked hypertension which was often overlooked by the conventional resting BP method prompts the high cardiovascular risk in adults (7). However, thresholds of exercise BP that could reveal high BP or underlying cardiovascular risk in childhood and adolescence remains unclear. In addition to revealing underlying high BP and cardiovascular risk, many factors such as cardiorespiratory fitness and body composition (fatness) have been demonstrated associated with exercise BP and could modify its association with the cardiovascular structure in adulthood. However, it is unknown whether this is the case in childhood and adolescence. By reviewing the current knowledge/literature, this review aims to achieve a better understanding of the factors that contribute to exercise BP (including fitness and fatness), and the association between exercise BP in childhood and adolescence and potential future cardiovascular risk (high BP and altered cardiovascular structure).

Although we have performed a series of studies to explore these associations in adolescence, (92–94) the existing literature on exercise BP in pediatric and adolescent populations is still scarce, lacking precise and valuable reference data for evaluating BP following various exercise protocols, as well as identifying potential clinical conditions that may contribute to an exaggerated BP response. Therefore, a deeper understanding the implications of such exaggerated BP responses is crucial in clinical practice.

Fitness and fatness and other factors is crucial in contributing to exercise BP and its association with the cardiovascular risk in childhood and adolescence. Deeper exploration into early-life exercise BP and its clinical utility in detecting future cardiovascular risk should be encouraged.

## Author contributions

ZH: Conceptualization, Writing – original draft, Writing – review & editing. XiuL: Writing – review & editing. XiaL: Writing – review & editing. YX: Writing – review & editing. HF: Writing – review & editing. LR: Writing – review & editing.
